# Liver Diseases: Science, Fiction and the Foreseeable Future

**DOI:** 10.3390/jpm14050492

**Published:** 2024-05-04

**Authors:** Robert K. Gieseler, Theodor Baars, Mustafa K. Özçürümez, Ali Canbay

**Affiliations:** Department of Medicine, University Hospital Knappschaftskrankenhaus, Ruhr University Bochum, 44892 Bochum, Germany; theodor.baars@t-online.de (T.B.); mustafa.oezcueruemez@kk-bochum.de (M.K.Ö.)

**Keywords:** history of hepatology, MASLD/NAFLD, liver fibrosis/cirrhosis, chronic viral hepatitides, liver transplantation, liver–microbiome axis, organokine crosstalk, chronobiology, liver aging

## Abstract

This Editorial precedes the Special Issue entitled “Novel Challenges and Therapeutic Options for Liver Diseases”. Following a historical outline of the roots of hepatology, we provide a brief insight into our colleagues’ contributions in this issue on the current developments in this discipline related to the prevention of liver diseases, the metabolic dysfunction-associated steatotic liver disease (or non-alcoholic fatty liver disease, respectively), liver cirrhosis, chronic viral hepatitides, acute-on-chronic liver failure, liver transplantation, the liver–microbiome axis and microbiome transplantation, and telemedicine. We further add some topics not covered by the contributions herein that will likely impact future hepatology. Clinically, these comprise the predictive potential of organokine crosstalk and treatment options for liver fibrosis. With regard to promising developments in basic research, some current findings on the genetic basis of metabolism-associated chronic liver diseases, chronobiology, metabolic zonation of the liver, aspects of the aging liver against the background of demography, and liver regeneration will be presented. We expect machine learning to thrive as an overarching topic throughout hepatology. The largest study to date on the early detection of liver damage—which has been kicked off on 1 March 2024—is highlighted, too.

## 1. Memorable Origins

Allow us to begin with a brief yet meaningful journey into the past before we embark on a voyage towards the future of personalized hepatology with this Special Issue. As a humble remark in advance, however, the space available here only allows for a few select brushstrokes taken from several wall-filling paintings of life.

Knowing our past is the prerequisite for being able to shape the future based on a well-aligned inner compass and a clear vision. So, who were the forefathers and foremothers of our discipline? While many faces and names may come to mind, five of them stand out clearly—and regrettably, it seems as if each new generation needs to be reminded time and again of the first and perhaps most trailblazing among them. There is an extremely malevolent reason behind this, which is must-tell history: just like so many other Jews, Ismar Isidor Boas (1858–1938) was inevitably exposed to the maelstrom of Nazi racial fanatism in 1930s’ Germany. If they had had their way, both he as a human being and his seminal œuvre should have been erased from the face of the earth. And, they almost succeeded: already in his seventies, Boas fled from Berlin to Vienna in 1936 in the wake of the ‘Nuremberg (Racial) Laws’, only to find himself exposed again to the threat of persecution after the Austrian *Anschluss* (annexation) to the so-called ‘Third Reich’. Three days after the Nazis entered Vienna in 1938, he took his own life [1]. The legacy of his work had already been taken away from Professor Boas by the professional society he had helped to shape so much: the *Deutsche Gesellschaft für Gastroenterologie, Verdauungs-und Stoffwechselkrankheiten* (i.e., the German Society for Gastroenterology, Digestive and Metabolic Diseases) (DGVS). Or so it seemed—but a crucial document had been preserved. As it says on the DGVS website, “*While doing research for the commemorative publication “100 Years DGVS” in 2013, the transcript of proceedings of the then Secretary General was discovered. It contained the society’s 1932/33 membership list*” [2]. There it was, a record revealing the names of its former Jewish members crossed out in red pencil and expelled—a third of all colleagues. Following this discovery, the DGVS initiated the project “Gegen das Vergessen” (literally “Against Forgetting”), entitled “We Remember” in its English-language version. It can be found on the society’s website and commemorates all of those “*Jewish physicians who were excluded from the specialist society, who were disenfranchised, persecuted, forced to flee Germany, or deported to concentration camps (…)*” [2]. With reference to Hoenig and Boyle, we would like to invite you to take a closer look at the fate of Ismar Boas: as early as 1988, they delved into his life, his work, and the circumstances that drove him to his death [1]. As far as our topic is concerned, Boas, at an age of less than thirty, founded the field of gastroenterology in 1886, which he shaped for over half a decade, in addition to numerous other achievements. Separating the field of gastroenterology from internal medicine—which he had to defend against considerable opposition—can in fact be considered a key prerequisite for the subsequent establishment of hepatology as yet another distinct discipline [1,2]. 

However, we can go back even further to the period from 1858 to 1861 when Friedrich Theodor Frerichs (1819–1885) laid the foundation for science-based hepatology with his two-volume opus ‘Clinic of Liver Diseases’ [3,4,5]. Accordingly, his successor Ernst von Leyden stated that Frerichs had transferred medicine to the ranks of the exact natural sciences [6]. Still, Frerichs was committed to maintaining the unity of internal medicine [5], and it took several more decades for hepatology to blossom as a separate field. In this respect, Hans Philipp Popper (1903–1988) [7,8] and Dame Sheila Patricia Violet Sherlock (1918–2001) [9,10] are reasonably assumed to be household names to the hepatological community as they were the ones who, in the 1940s, ‘surgically resected’ the new discipline of hepatology from the corpus of gastroenterology [7,8,9,10].

Finally, one might arguably include Nancy Leslie Rutherford Bucher (1913–2017) as another founding mother in this illustrious circle [11]. In 1964, she was the first to show that the most striking effect of aging on the liver is the decline of the organ’s remarkable capacity to ‘regenerate’ (a process correctly termed ‘compensatory hyperplasia’) in the event of tissue loss [12]. Not only epistemologically, but also in terms of practical medical relevance, this decline must be viewed against an evolutionary background spanning eons that helps to explain aging-related hepatic and systemic pathological effects, which may lead to novel options for their antagonization [11,13].

Mirroring the progress of knowledge, hepatology has thus repeatedly shed its skin. In more recent times, the liver has increasingly been shown to intersect with other organs and systems, typically manifesting as “axes”—such as the gut–liver axis, the brain–liver axis, the gut–brain–liver axis, and the liver–microbiome axis—or, for example, reflected in the form of organokine crosstalk. Still, hepatology has not continued to divide into further sub-disciplines, but today consists of various subspecialties, some of which are featured in this Special Issue. It is probably correct to say that hepatology would not be what it is today had it not been for the luminaries briefly sketched above. They created the discipline’s cornerstones. Let us try to prove ourselves worthy successors to these pre-eminent colleagues and their groundbreaking achievements with the perspectives presented in this Special Issue and with our future endeavors in hepatology.

## 2. Back to the Future: Forward-Looking Topics in This Special Issue

We will now briefly outline the contributions to this Special Issue and discuss some previous publications in the respective contexts.

### 2.1. Prevention of Liver Diseases

In this Special Issue, Muñoz-Restrepo et al. highlight successfully implemented measures for preventing liver diseases, such as vaccination strategies, novel medications, lifestyle changes, and preventive surgeries. However, they also point to the parallel worldwide increase in chronic liver diseases—prominently including metabolic dysfunction-associated steatotic liver disease (or non-alcoholic fatty liver disease, respectively) (MASLD/NAFLD) (cf. Section 2.2.) as well as chronic hepatitis B and hepatitis C (cf. Section 2.4.)—which collectively call for better prevention strategies that may also result from big-data analyses [14]. This will likely create options for personalized medicine.

### 2.2. MASLD/NAFLD

The MASLD/NAFLD pandemic, which affects >25% of humanity, is the greatest hepatological challenge. It can still only be treated inadequately, so dietary and behavioral measures are currently the best options available [15]. Following the initial suggestion by Eslam and colleagues on renaming NAFLD to MAFLD [16,17], we are now trending towards the term (see above) and acronym MASLD. Ultimately, this renaming will result in the review and update of the nomenclature and subphenotypes of this condition, which could impact personalized medicine interventions for patients suffering from this disease entity.

In this Special Issue, Kreimeyer et al. show that the treatment of patients with the severe inflammatory form of MASLD/NAFLD (i.e., NASH) with bile acid transporter (BAT) gene polymorphisms in ABCB4 or ABCB11 with ursodeoxycholic acid for 12 months significantly reduced GGT (all patients) and ALT (homozygous patients). Patients with the TM6SF2 polymorphism showed a significant reduction in both GGT and ALT. Thus, NASH patients with elevated GGT should be screened for BAT gene polymorphisms prior to treatment [18]. 

Also, in this Special Issue, Schwertheim et al. examined selected protein expressions in liver tissue from MASLD/NAFLD patients to clarify their potential alteration in the progression from simple steatosis to NASH. The expression of pNRF2, SOCS3, and RIG1 (hepatocytes), and (bile ducts) was significantly higher in NASH than in steatosis; thus, these proteins might be assessed as potential therapeutic targets [19]. 

### 2.3. Liver Cirrhosis

Rotational thromboelastometry is a viscoelastic method that allows to quickly assess the state of induced hemostasis in whole blood samples and to effectively reduce the number of transfusions, healthcare costs, and complications [20]. The pressure of blood flowing through a cirrhotic liver can be reduced via the placement of a transjugular intrahepatic portosystemic shunt (TIPS) and is employed for treating refractory ascites and/or variceal bleeding [21].

In this Special Issue, Bedreli et al. evaluated alterations and differences in coagulation in the portal and peripheral circulation (PORC; PERC) via rotational thromboelastometry during TIPS. In blood samples from a cohort of cirrhotic patients (MELD Score: 12; median age: 60 years) undergoing TIPS, the authors detected no coagulation differences between PERC and PORC, which contrasts previous reports that suggested increased clotting activity in PORC vs. PERC in patients with liver cirrhosis [22]. 

### 2.4. Chronic Viral Hepatitides

The viral hepatitides remain a major public health problem, with five biologically unrelated hepatotropic viruses responsible for the majority of the global burden. The highest numbers of chronic infections and deaths alike are due to the hepatitis B and hepatitis C viruses (HBV; HCV), with approximately 257 million or 71 million infected people, respectively [23]. However, infection with the hepatitis D virus (HDV)—which occurs in association with HBV—also affects between 12 and 72 million people worldwide, and it is associated with a more rapid progression to cirrhosis and liver failure as well as higher rates of hepatocellular carcinoma than infections with HBV or HCV alone [24].

In this Special Issue, Schlaak gives an overview of the current treatments and developments, stating that (i) the introduction of direct-acting antivirals for treating HCV has been a boon by all accounts (at least, one might add, for those patients living in countries where these drugs are available); (ii) nowadays, HBV is generally well controllable, although a “functional cure” has not yet been achieved, which calls for novel therapeutic strategies that are currently being developed; and (iii) HDV remains the most challenging type of chronic viral hepatitis, for which therapeutic approaches with better response rates must be conceived [25]. 

### 2.5. Acute-on-Chronic Liver Failure

Acute-on-chronic liver failure (ACLF) is a condition in patients with known chronic liver disease and acute decompensation (AD) of liver cirrhosis. This syndrome is characterized by severe systemic inflammation and proinflammatory precipitating events (such as infections), and it is associated with single or multiple organ failure. Therefore, patients with ACLF are at a high risk of death within 28 days after hospital admission [26].

In this Special Issue, Kimmann et al. present the pathophysiological and clinical background to AD and ACLF as well as the current interventional treatment options—with liver transplantation (LTx) as the only curative treatment option currently available—and they further expand on future therapeutic options for ACLF management of AD as well as of ACLF [27]. 

### 2.6. Liver Transplantation

Apart from ACLF (cf. Section 2.5), another indication for LTx comprises neuroendocrine tumors (NETs) with liver metastases, although this rare indication consists of only <1% of all LTx activity. Specifically, favorable and acceptable transplant candidates are NET patients with metastases that are confined to the liver, are not poorly differentiated, are non-resectable, and are treatment-resistant [28]. 

In their narrative systematic review on this therapeutic approach, which is still controversially debated, Palaniappan et al. critically appraise the existing literature regarding this modality and thus provide an important basis for further discussing the role of LTx in the setting of patients suffering from NETs with liver metastases that meet the required criteria [29].

### 2.7. Liver–Microbiome Axis and Gut Microbiome Transplantation

An ever-increasing body of knowledge clearly shows that the gut microbiome, dietary habits, and metabolic health (or, conversely, metabolic diseases) form closely associated functionally interactive intersections. More recently, it has become apparent that the gut microbiota functions as a mediator of the dietary impact on the metabolic status. Against this background, causal relationships are increasingly being elucidated, which may let us therapeutically address metabolic diseases by personalized nutrition in the future [30]. 

Another approach for treating metabolic diseases is fecal microbiome transplantation (FMT), which is dealt with by Stadlbauer in this Special Issue. Although differing from the approach of personalized nutrition, its intention is identical in that it aims at altering a patient’s microbiome composition. However, FMT is presently approved for proof-of-concept studies only and is not yet ready for a broad application. Against this background, Stadlbauer presents an overview of the current knowledge and describes the tasks ahead to be tackled for making FMT available to larger patient populations [31]. In this regard, methodological advances and standardization approaches, such as the one just published by Lederer and colleagues, will certainly be instrumental [32].

### 2.8. Telemedicine for Remote Monitoring

Telehealth or telemedicine—i.e., remote electronic patient monitoring as well as the provision of medical information and services via telecommunication—has considerably increased during the SARS-CoV-2 pandemic [33]. 

Accordingly, Akbar et al., in this Special Issue, assess the role of telemedicine in monitoring MASLD/NAFLD during the pandemic. Their systematic review and meta-analysis demonstrate that telemedicine via mobile applications during the SARS-CoV-2 pandemic proved to be an option for monitoring lifestyle modifications in MASLD/NAFLD patients [34].

In 2022, Greiwe surmised that telemedicine will remain an effective tool in the future, regardless of its use during the pandemic [33]. This assumption is, for instance, confirmed by the situation in Germany, where telemedicine networks are currently being expanded in all federal states against the backdrop of the country’s continuously increasing demographic challenges with rising care and treatment needs, while the staffing levels are chronically low [35]. 

## 3. Further Forward-Looking Topics

We will now provide a brief overview of some other future-oriented topics in hepatology not covered by articles featured in this Special Issue. This subjective non-exhaustive selection reflects some of the aspects that we believe may become increasingly important with respect to clinical applications, clinical trials, and clinically oriented basic research.

### 3.1. Clinical Opportunities: The Foreseeable Future

#### 3.1.1. Predictive Potential: Organokine Crosstalk

MASLD/NAFLD increases the risk for cardiovascular disease (CVD) [36]. However, despite the high numbers of MASLD/NAFLD cases, the medical need for a reliable scoring system to predict the CVD risk remains unmet. We recently presented cumulative evidence supporting the assumption that this may be achieved by establishing an algorithm based on the systemic release of organokines, whose healthy pattern changes in disease. Specifically focusing on adipokines, hepatokines, and cardiokines, we thus hypothesized that an algorithm predictive of the CVD risk in patients with MASLD/NAFLD can be established and improved continuously via machine learning. Once implemented, such a score might be used to estimate the risk for CVD for prevention and early stage life-saving interventions [15]. In general, we venture that computing the diverse and complex data through machine learning will ultimately yield excellent applications for personalized medicine, which will likely extend even beyond the fields of hepatology and cardiology.

#### 3.1.2. Therapeutic Potential: Vitamin D in Liver Fibrosis

We previously showed that vitamin D (VD) may be a treatment option early in the onset of liver fibrosis in patients with certain VD receptor (VDR) genotypes or VDR polymorphisms. Specifically, targets within the TGF-β pathway might provide opportunities for patients with detrimental VDR single-nucleotide polymorphisms [37]. In a systematic review on the benefit of VD supplementation, Sharifi and Amani highlighted partly contradictory findings between different clinical trials and pointed out that influencing factors, such as gender, VD co-supplementation with calcium, and gene polymorphisms, should be considered in future clinical studies [38]. The clear VDR genotype dependencies we already emphasized [37] may explain some of those contradictory clinical trial results. We therefore maintain our view that VD supplementation could be a future treatment option early in the onset of liver fibrosis, provided that the treated patient cohort is strictly defined.

### 3.2. Basic Research: What Is on the Horizon?

#### 3.2.1. Predictive Potential: Genetic Underpinnings of Major Liver Diseases

Only very few studies into the genetic underpinnings of major liver diseases go back in time as far as a most recently published evolutionary investigation. Herein, a common variant—i.e., the risk allele *PNPLA3* p.I148M (rs738409)—of the gene for patatin-like phospholipase domain-containing 3 (PNPLA3), which is prominently associated with an increased risk to develop steatotic liver disease, MASLD/NAFLD (including progressive inflammation), liver cirrhosis, and hepatocellular carcinoma [39], was studied. Contrary to non-human primates, this risk allele was identified in all Neanderthals and Denisovans, which indicates that the risk allele emerged prior to the split between the Neanderthals and modern humans. Interestingly, however, present-day humans exhibit a wide range (i.e., 8% to 72%) of the presence of *PNPLA3* p.I148M, depending on their ethnicity [40]. The authors may thus have identified the earliest evolutionarily known risk gene for developing certain severe liver diseases, but it is too early to assess whether and how this knowledge may be used in the future.

#### 3.2.2. Predictive Potential: Early Detection of Liver Damage

Particularly in this Special Issue, which has a focus on future hepatology, we should not forget to mention that the project “A Biomarker-Based Platform for Early Diagnosis of Chronic Liver Disease to Enable Personalized Therapy”, funded by the European Commission with a volume of EUR 15 million and designed for five years, was launched on 1 March 2024 [41]. This consortium involves 23 academic and industrial partners from 11 European countries, including Croatia (1), Denmark (3), France (1), Germany (6), Ireland (1), Italy (2), Slovakia (1), Spain (4), Sweden (1), and Switzerland (1), as well as the transnational European Liver Patients’ Association (ELPA) located in Brussels, Belgium, with overarching support from Innovation Acta headquartered in Rome, Italy. This is the world’s largest study to date on the early detection of liver damage—an immensely important field of research, particularly in view of the fact that, unlike other organs, the damaged liver often “complains” about its deplorable condition too late. This program is designed to detect liver fibrosis at an early stage to prevent the development of cirrhosis and liver cancer. We believe that medicine, science, and, first and foremost, the patients can look forward to the results of this project. 

#### 3.2.3. Therapeutic Potential: Metabolic Syndrome and MASLD/NAFLD

As mentioned, treatment options for metabolic diseases—and MASLD/NAFLD in particular—are extremely limited. Therefore, this topic is being intensively researched. We recently presented a study on the application of l-ornithine–l-aspartate (LOLA) (the stable salt of l-ornithine and l-aspartic acid) in human in vitro models of steatosis, insulin resistance and metabolic syndrome [42]. In addition to the known effects of LOLA on NH_3_ detoxification (e.g., [43]), this agent normalized fatty acid transport regulation, branched-chain amino acid catabolism, energy consumption, and mitochondrial energy balance. We thus suggested that this relatively inexpensive active agent may significantly contribute to a safer, more effective, and gentler management of metabolic diseases, including MASLD/NAFLD [42].

#### 3.2.4. Hepatic Chronobiology

To our knowledge, the first paper on a chronobiological aspect of the liver was published in 1981 [44]. Yet, this comparatively young sub-discipline has great future potential. Regarding the role of chronobiology in metabolic disease, Amatobi and colleagues have summed up a piece of fundamental insight in a wonderful sentence: “*Modern lifestyle is often at odds with endogenously driven rhythmicity, which can lead to circadian disruption and metabolic syndrome*”. In a trailblazing study performed in *Drosophila melanogaster* (a well-established model in chronobiology), the authors elucidated several important relationships between metabolite cycling and the metabolic status, the disruption of circadian rhythmicity, and the propensity for metabolic disease [45]. Thus, while fruit flies and humans admittedly are separated by a large evolutionary gap, such relationships have long been established in the living world, allowing us to learn more regarding hepatic metabolic diseases in humans. Although not immediately implementable in the clinic, studies like this one showcase the great potential of basic research for yielding new medical approaches in the more distant future.

#### 3.2.5. Metabolic Zonation of the Liver

More than 45 years ago, Jungermann and Sasse introduced the model of metabolic zonation of the liver, on which today’s understanding of a dynamically organized organ with functionally specialized hepatocyte variants is based [46]. With his integrative view, Jungermann was also among the first to recognize the important role of neuronal and inflammatory signaling in the regulation of the liver’s metabolic functions [47]. We presume that the spectrum of implications of these important aspects of hepatic versatility is far from being fully explored. For example, the integration of metabolic zonation with chronobiological, age-related, and pharmacogenomic aspects will add new dimensions of complexity that should best be analyzed by intelligently programmed machine learning to improve disease prevention measures and optimize the treatment of chronic and malignant liver diseases.

#### 3.2.6. Liver Aging and the Aging Societies

As early as 1985, Hans Popper (see above) farsightedly predicted that “*the effect of age on the liver and of the liver on aging is full of promise if available methodologies are rigorously applied*” [48]. Such promise meets urgent societal demand: already covering an extended period of time and clearly extrapolated into the future, demographics show an increasing trend towards population aging, which is not limited to the Western societies’ Baby-Boomer issue [49]. In fact, the World Health Organization has predicted that the global proportion of people >60 years of age will increase from 12% to 22% between 2015 and 2050 [50]. Therefore, as the body of knowledge about the liver and aging, as well as the available methodologies, have increased considerably in recent decades, they can—and definitely should—now be applied rigorously to minimize the negative health effects and their social and economic implications. This includes all hepatic aspects of aging covering genomics and epigenomics [48], age-related changes of the hepatic transcriptome [51], dietary habits in conjunction with medically based guidelines for the food industry vis-à-vis the continuous increase in metabolic disorders [52], and, last but not least, important evolutionary underpinnings of hepatic biology that go along with an increased likelihood for developing certain chronic diseases, while chronic diseases in turn accelerate biological aging [11]. We therefore have an impressive “toolbox” at our disposal. It is now up to healthcare policies to set the necessary course: given our immense challenges, it would be inexcusable to shirk this responsibility.

#### 3.2.7. Liver Regeneration

The liver’s ability to regenerate after tissue loss [11,13] will always remain a central topic in many hepatological contexts. We can expect further fascinating findings in this area that will help expand our medical use of this capacity. Here is a prime example: hepatocyte membrane-specific phospholipids (i.e., short-chain fatty acids) synthesized by gut microbiota and delivered via the gut–liver axis have been demonstrated to promote liver regeneration. The authors concluded that this process is pivotal for hepatic compensatory hyperplasia [53]—and in their Editorial Commentary on this article, Jian et al. stated that “*the revolutionary clinical value of postoperative interventions based on gut microbiota in patients undergoing liver surgery will undoubtedly propel gut microbial interventions to become a standard of care in the future*” [54].

### 3.3. Machine Learning—An Overarching Aspect in Hepatology (and Beyond)

We already repeatedly mentioned the use of machine learning (ML) in various hepatological contexts. ML [55] is defined as a subset of artificial intelligence [56]. It has experienced rapid growth in all fields of research and application, including the medical area, where speed, accuracy, and efficiency have steadily improved. Therefore, as a result of data collection, analysis, and classification, ML has continuously enhanced the prediction of various diseases [55]. Our own experience with the application of ML has been very encouraging [57,58,59,60]. We further believe that computation using suitable ML techniques will enable us to establish algorithms that will permanently learn from the acquired data via probabilistic modeling [61]. All of those interested may find an excellent review and critical appraisal on the application of ML in hepatology by Spann et al. to be instructive [62]. Overall, while some desirable applications may remain a fiction in the near future, ML will undoubtedly play a very important role in hepatology—not to mention its hard-to-overestimate potential for personalized medicine as a whole.

## 4. Epilogue

**Figure 1:**
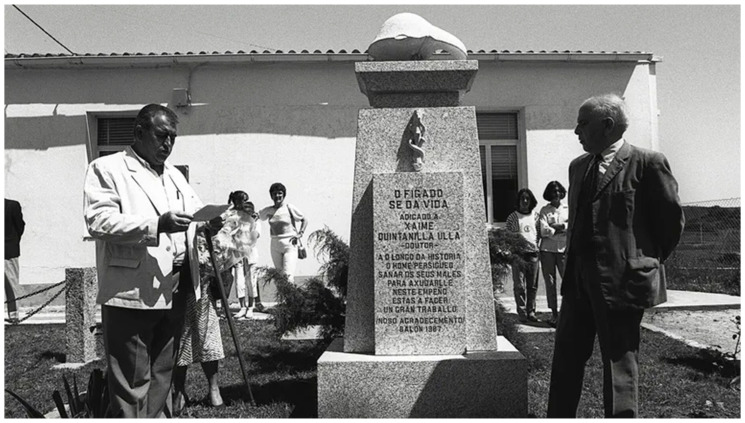
Xaime Quintanilla Ulla, M.D., dedicates the liver monument in 1987. The translated inscription reads “The liver gives life” (photo credit: Rodrigo R. Arda [63]).

Working tirelessly and altruistically in the organism’s engine room, the liver is constantly exposed to all our impositions—be it gluttony, the abuse of alcohol, illegal drugs or medications, and the challenge of polypharmacy. Trying to protect us from mischief, it stands solid as a rock. However, nobody takes notice—this organ does not complain; the liver just perishes quietly when it can take no more. Similar thoughts might have crossed the mind of Dr. Xaime Quintanilla Ulla, who, as a physician and mayor of the Spanish municipality of Ferrol, declared to the local council in 1987, “*Al hígado habría que hacerle un monumento*” (“The liver should be given a monument”). So, in the same year, a monolith was erected in honor of this organ (Figure 1); it remains the only liver monument in the world to this day [63].

This man had definitely recognized the preciousness of this remarkable organ. Given his medical background, this project was a matter close to his heart—and the liver truly deserves its monument. Not only may the rapidly increasing options of personalized medicine enable us to improve the management of chronic liver diseases, but with the liver’s central role being closely intertwined with all bodily aspects, it eventually could also be key to the implementation of life-prolonging measures.

We hope you find this Special Issue a pleasant and insightful read.
